# Developing a standard definition for sequences of concern

**DOI:** 10.3389/fbioe.2026.1830842

**Published:** 2026-06-04

**Authors:** Tessa Alexanian, Jacob Beal, Craig Bartling, Jens Berlips, Peter A. Carr, Adam Clore, Helena Cozzarini, James Diggans, Yorgo El Moubayed, Kevin Esvelt, Kevin Flyangolts, Leonard Foner, Patrick A. Fullerton, Bryan T. Gemler, Caitlin A. D. Jagla, Rassin Lababidi, Tom Mitchell, Steven T. Murphy, Michael T. Parker, Nicholas Roehner, Andre Rusch, Kemper Talley, Troy Timmerman, Nicole E. Wheeler

**Affiliations:** 1 International Biosecurity and Biosafety Initiative for Science (IBBIS), Geneva, Switzerland; 2 RTX BBN Technologies, Cambridge, MA, United States; 3 Battelle Memorial Institute, Columbus, OH, United States; 4 SecureDNA, Basel, Switzerland; 5 Integrated DNA Technologies, Coralville, IA, United States; 6 Twist Bioscience, South San Francisco, CA, United States; 7 Massachusetts Institute of Technology, Cambridge, MA, United States; 8 Aclid, New York, NY, United States; 9 College of Arts and Sciences, Georgetown University, Washington, DC, United States; 10 Thermo Fisher Scientific, Regensburg, Bayern, Germany; 11 University of Birmingham, Birmingham, United Kingdom

**Keywords:** biosecurity, DNA synthesis, gene synthesis, nucleic acids, screening, sequences of concern, standards

## Abstract

Readily available nucleic acid synthesis is both critical for the bioeconomy and an increasingly pressing security concern due to the potential for accidental or deliberate misuse. While biosecurity experts broadly agree that nucleic acid providers should screen orders for potential “sequences of concern,” there has previously been no agreed standard for how to define and recognize such sequences. To address this gap, we first organized a collection of test sets containing 1.1 million sequences from pathogens and toxins on the Australia Group Common Control Lists and their non-controlled relatives, along with model organisms and synthetic constructs. An initial categorization of sequences as to whether or not they were sequences of concern was produced by comparing the results of four biosecurity screening systems for each of these sequences, finding that these systems already agreed on the categorization of more than 80% of sequences. We then refined these results through a science-based stakeholder review process to define a rubric for determining whether a sequence should be flagged as a potential sequence of concern, then applied this rubric to improve the categorization of sequences in test sets. The result is a rubric that identifies sequences of concern with respect to human pandemic-potential viruses, key classes of low-risk genes, and controlled toxins. Applying this rubric to the test set collection has, to date, reduced the number of test sequences with disputed categorization by 44.3% for controlled viruses and 10.7% across the collection of test sets as a whole. Together, the rubric and the test sets provide a concrete “sequence of concern” definition that can be used as a foundation for development of biosecurity screening standards and policy and is also continuing to be refined in ongoing work.

## 1 Introduction

Readily available nucleic acid synthesis is both a critical supply chain input for the bioeconomy and an increasingly pressing security concern, given the potential for accidental or deliberate misuse of genes for dangerous pathogens or toxins (Hoffmann et al., 2023; Diggans and Leproust, 2019). There is broad consensus amongst biosecurity stakeholders that one of the key defenses against such misuse is screening of nucleic acid orders for sequences of concern (World Health Organization, 2022; National Academies of Sciences, Engineering, and Medicine, Division on Earth and Life Studies, Board on Life Sciences, Board on Chemical Sciences and Technology, Committee on Strategies for Identifying and Addressing Potential Biodefense Vulnerabilities Posed by Synthetic Biology, 2018; International Gene Synthesis Consortium, 2024; International Organization for Standardization, 2024). Requirements for nucleic acid order screening have begun to be incorporated into biosecurity policy, including in both the United States (The White House Office of Science and Technology Policy OSTP, 2024; The White House, 2025) and the UK (UK Department for Science, Innovation, and Technology, 2024).

A key open question, however, has been how to determine which sequences should be considered “sequences of concern” and thus trigger an evaluation of the customer to determine whether the sequences should be provided or denied and whether the attempted acquisition should be reported to appropriate regulatory or law enforcement authorities. Agreement upon a shared standard definition for sequences of concern is necessary in order to evaluate the efficacy of screening methods, which is in turn required in order for screening requirements to be effective and enforceable (Wheeler et al., 2024a; Laird et al., 2025; Millett et al., 2023). Moreover, the definition for sequences of concern must not just be theoretical (e.g., “virulence factor” or “capable of endowing or enhancing pathogenicity”) but also *operational* in the sense that it enables different organizations to make equivalent decisions across a wide range of specific pathogen and toxin genes.

Accordingly, the Sequence Biosecurity Risk Consortium (SBRC) was founded to address this need by bringing together synthesis providers, screening tool developers, policymakers, and scientific experts to create and maintain a science-based definition for sequences of concern (Beal and Alexanian, 2025). The SBRC grew directly out of the International Gene Synthesis Consortium’s (IGSC) Test Set Working Group, which developed the multi-tool comparison methodology used here to develop draft categorizations (Wheeler et al., 2024a). The IGSC also maintains the Regulated Pathogen Database (International Gene Synthesis Consortium, 2024) that serves as the taxonomic foundation for how the work reported in this manuscript organizes sequences into test sets. This evolution from an industry-led working group to a broader multi-stakeholder consortium reflects the IGSC’s longstanding role in establishing biosecurity screening as an industry norm, now formalized into a process capable of supporting enforceable standards.

Here we report on the first key results from the SBRC’s effort to develop a standard “sequence of concern” definition: development of an operational definition in the form of a large-scale collection of sequence test sets and a complementary theoretical definition in the form of a rubric for categorization of sequences. We first generated a large-scale collection of test sets comprising approximately 1.1 million sequences from pathogens and toxins on the Australia Group Common Control Lists and their non-controlled taxonomic relatives, along with model organisms and synthetic constructs. We next determined the initial categories of test set sequences through comparison of tool results using the methodology established in our prior work (Wheeler et al., 2024a), finding an overall agreement on the categorization of more than 80% of test sequences. These results were then refined through a science-based stakeholder review process adapted from the community standards development processes used by the IETF (Resnick, 2014), Python (Warsaw et al., 2000), and SBOL (SBOL Community, 2025) communities.

The product of this work, reported herein, is the SBRC Screening Testing Collection version 1.0, consisting of both rubric and test sets. The version 1.0 rubric defines sequences of concern with respect to human pandemic-potential viruses, key classes of low-risk genes, and controlled toxins. Application of that rubric to the test sets has produced the version 1.0 test set collection, reducing the number of test sequences with disputed categorization by 44.3% for controlled viruses and 10.7% across the test set collection as a whole. Together, the version 1.0 rubric and the improved test sets provide a concrete “sequence of concern” definition that can be used as a foundation for development of biosecurity screening standards and policy, while ongoing work continues to apply these same methods to extend the definition (e.g., to high-risk bacterial pathogens) for further versions of the SBRC Screening Testing Collection.

In the remainder of this paper, we first detail the construction of the collection of test sets and initial categorization of sequences in Section 2, then describe the refinement process and its results in Section 3. Finally, Section 4 summarizes these results and discusses how they can be used to support enforceable biosecurity screening requirements, as well as discussing ongoing efforts in the SBRC and needs for complementary standards development.

## 2 Test set construction and initial categorization

We began the process of defining sequences of concern with an empirical approach, previously prototyped in Wheeler et al. (2024a), in which *de facto* areas of agreement and dispute are discovered by running sequences through multiple biosecurity tools and comparing their results. Where comparison of results finds agreement, the categorization of sequences can be used as a first approximation of an operational “sequence of concern” definition (e.g., in *Bacillus anthracis* the “lethal factor” gene is high risk, while its ribosomal RNA genes are low risk). While agreement does not necessarily indicate a correct assessment of risk, areas of dispute are likely to be more important to resolve in order to ensure that dangerous orders can be consistently identified. We thus first constructed a large-scale collection of test sets of nucleic acid sequences intended to enable comprehensive testing, then evaluated all test set sequences in four biosecurity screening tools, and finally analyzed the results of this initial categorization as a candidate empirical definition of sequences of concern.

### 2.1 Test set construction

We began by scaling up the methodology used in Wheeler et al. (2024a) to construct a collection of test sequences covering the full range of pathogens and toxins in the IGSC Regulated Pathogen Database (International Gene Synthesis Consortium, 2024). The IGSC RPD covers all regulated biological agents listed on the Australia Group Common Control Lists, the US Export Administration Regulation Commerce Control List, the US Federal Select Agent Program, and the European Union list of dual-use items, comprising 39 taxonomic clusters of regulated pathogens, plus additional lists for regulated toxins.

For each taxonomic cluster, test sets were constructed for both regulated threats (i.e., pathogenic organisms and toxins) and closely related relatives. For example, the *Bacillus cereus group* cluster contains threat taxa *B. anthracis* and *B. cereus biovar anthracis* and non-regulated relatives such as *B. cereus*, *Bacillus thuringiensis*, and *Bacillus toyonensis*. Test sets were constructed for nucleic acid sequences and reverse-translated protein sequences, each comprising up to 10,000 sequences in NCBI between 200 bp and 10,000 bp in length from the relevant taxa. In cases where more than 10,000 sequences were available, the number was reduced by taking a random subset of the available NCBI sequences.

Exceptions were made in the following cases: viroids do not express proteins, and thus have no protein test sequences; the *Peronosporaceae* cluster has no protein threat test sequences because NCBI currently contains no protein sequences besides a few highly conserved genes; and the toxin collections use additional filtering to remove non-toxin genes.

Finally, two additional clusters of low-risk sequences were added to address common sources of false positives. One cluster contains 10,000 bp sequences taken from the genomes of a diverse collection of model organisms, as model organisms are common subjects of research and development and thus their genes are frequently found in nucleic acid orders. Genomes used for generating this collection include *Homo sapiens* (humans), *E. coli* (bacteria), *Arabidopsis thaliana* (plant), *Tetrahymena thermophila* (ciliated protozoan), *Dictyostelium discoideum* (slime mold), *Saccharomyces cerevisiae* (yeast), *Drosophila melanogaster* (fruit fly), *Danio rerio* (fish), and *Chlamydomonas reinhardtii* (single-cell algae). The second cluster represents engineered sequences, and was constructed using nucleotide sequences sampled from the iGEM registry of standard biological parts (iGEM Foundation, 2026) and filtered to contain only sequences between 200 bp and 10,000 bp in length. These sequences contain many frequently reused genetic tools, such as fluorescent reporters, purification tags, gene editing nucleases, and constitutive promoters, the vast majority of which (though not all) are low risk.

The result is a collection of approximately 1.1 million sequences divided into 42 clusters organized into four “kingdom-level” categories: 13 viral clusters, 17 bacterial clusters, 7 fungal clusters, and 5 other clusters (e.g., toxins, model organisms).

### 2.2 Screening tool comparison

**FIGURE 1:**
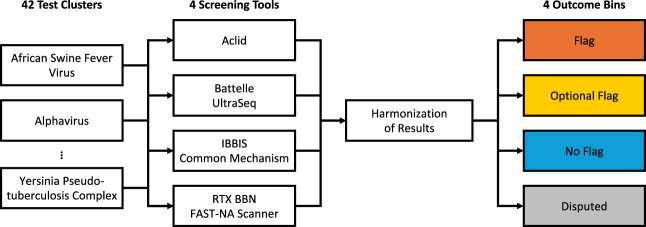
Test set sequences were assigned to initial categories by evaluating the sequences of each cluster independently with four biosecurity screening tools, then harmonizing results to categorize each sequence into one of four categories of consensus screening outcome. Figure adapted from Wheeler et al. (2024a).

To produce an initial categorization for each sequence in each test set, we applied the workflow from Wheeler et al. (2024a), as shown in Figure 1. Each sequence was independently screened by four biosecurity screening systems: Aclid (Aclid Inc, 2026), Battelle UltraSEQ (Gemler et al., 2023), the IBBIS Common Mechanism (Wheeler et al., 2024b), and RTX BBN FAST-NA Scanner (Beal et al., 2021; Wyschogrod et al., 2022), each applying is own distinct and independently developed algorithmic approach to determining whether to flag a sequence as a potential sequence of concern.

**FIGURE 2:**
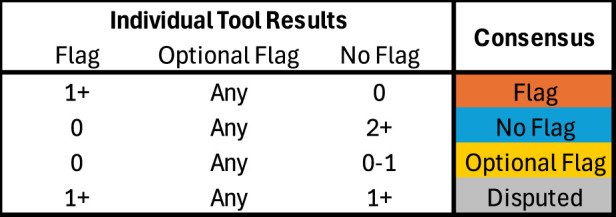
The initial category assigned to a test set sequence is determined by comparing the results of individual tools following the decision logic shown in this table.

The heterogenous reports produced by the four tools are then harmonized. First, each tool’s result for a given sequence is mapped into one of three general categories to describe a screening result (“Flag”, “No Flag”, or “Optional Flag”). The results of the individual tools are then combined following the decision logic shown in Figure 2 to place the sequences into one of four categories regarding consensus on screening outcome:

- Flag: sequence is “guilty” of being a sequence of concern (i.e., agreed to be potentially dangerous, such that an order containing that sequence should be flagged for follow-up screening): flagged by at least one tool and not cleared by any tool.
- No Flag: sequence is “innocent” of being a sequence of concern (i.e., a low-risk sequence that should not be flagged): cleared by at least two tools and not flagged by any tool.
- Optional Flag: sequence is “not guilty” and “not innocent” (i.e., not enough evidence to decide on a category): cleared by zero or one tool and not flagged by any tool.
- Disputed: sequence has a “hung jury” of tools disagreeing on biosecurity risk: flagged by at least one tool and cleared by at least one tool.

Note that the “Disputed” category was previously termed “Undetermined” in Wheeler et al. (2024a), but has been relabelled to better reflect how sequences in the category are being interpreted.

### 2.3 Results of initial categorization

**FIGURE 3:**
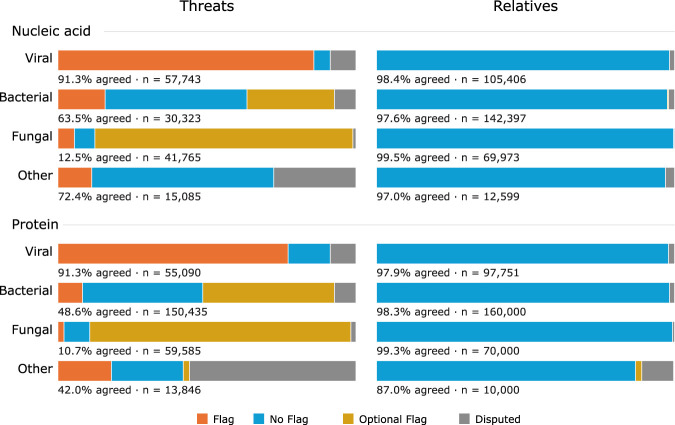
Results of initial categorization of test set sequences, with the “Agreed” category being defined as the sum of “Flag” and “No Flag” results. Exact values and combined statistics are provided in Supplementary Material: “Test Set Statistics.”

Figure 3 shows the results of the initial categorization of test set sequences produced by comparing the results of screening tools. Overall, the rate of agreement is high, with tools achieving either a “Flag” or “No Flag” consensus on 80.5% of all test set sequences. The rate of agreement is far higher for sequences from test sets for relatives than for sequences from test sets for threats, but even for threats there is agreement on the majority of test set sequences (52.8%). Importantly, the lower rate of agreement for threats is driven primarily by “Optional Flag” sequences for cellular threats (particularly fungi) rather than by “Disputed” sequences.

This is consistent with the prior results in Wheeler et al. (2024a) and with the observation that even in highly dangerous cellular pathogens such as *B. anthracis* or *Yersinia pestis*, most genes have little or nothing to do with what makes the organism a threat (Godbold et al., 2025). Moreover, many genes in such organisms are currently too poorly understood to make a meaningful assessment of potential risk, and thus also generally likely to be too poorly understood to be readily misused. Only 4.4% of test set sequences are “Disputed”: 8.5% of threats and 1.73% of relatives. Among relatives, some sequences were indeed flagged, but every such sequence was cleared by at least one other tool and thus ended up “Disputed”.

At the level of kingdoms, results for threats are again consistent with the prior small-scale test in Wheeler et al. (2024a). Amongst the threat sequences, viruses have the highest level of agreement, bacterial sequences are intermediate, and fungal sequences are dominated by poorly understood “Optional Flag” genes. The highest levels of disputes are around certain classes of toxins for which there are disagreements between tools on how to define risk. For bacteria, disputes are driven primarily by the interpretation of genes, while for viruses disputes are driven more by the interpretation of taxonomy. For example, when considering the controlled bacterial species *Rickettsia prowazekii*, tools disagreed on whether the PleD response regulator gene should be considered a virulence factor. On the other hand, when considering viral taxa in *Bunyavirales*, tools disagreed on whether Araraquara virus should be considered high risk based on its relationship to Andes virus.

Results for relatives are, as one would expect, consistently nearly all “No Flag”, with the exception of certain classes of toxin. Results for nucleic acid sequences and reverse-translated protein sequences are generally consistent with one another. The largest differences between these are primarily a statistical effect of the difference in numbers of sequences between test sets. Specifically, for bacteria the statistics are biased by the fact that poorly understood regions of bacterial genomes tend to appear in databases as large nucleic acid sequences annotated with many protein sequences, thus greatly enriching the rate of protein “Optional Flag” sequences relative to nucleic acid “Optional Flag” sequences in bacteria. This effect does not show up in viruses, where the number of genes is typically much smaller, or in eukaryotes, where databases tend to include mRNA transcripts. Meanwhile, statistics in the “other” category are affected by several clusters containing only nucleic acid sequences or only reverse-translated protein sequences.

All told, these results show that there already is a high degree of consistency between the implicit definitions of sequences of concern that are implemented by the biosecurity screening tools used to produce this initial categorization of sequences. At the same time, there is no inherent authority in the judgements of these particular tools. These results thus provide a starting point for building a definition of sequence of concern, which can then be complemented with a science-based approach to identifying and correcting errors and resolving disputes in the categorization of test set sequences.

## 3 Stakeholder-driven refinement of definitions

The artifacts produced by the SBRC complement the “bottom up” definition of sequence of concern in the test sets with a “top down” definition in the form of the Biosecurity Flag Rubric, a structured process for determining whether to flag a given nucleic acid or protein sequence. Applying the Biosecurity Flag Rubric to any sequence produces one of three results: “Flag”, “No Flag”, or “Undefined.” An “Undefined” result from the rubric, however, unlike the “Disputed” category for test set sequences, does not imply anything about the expected behavior of screening tools: it is simply that the clauses defining the rubric have not yet been extended to cover that particular class of sequence. The test sets and the Biosecurity Flag Rubric are linked together in a feedback loop that drives improvement of both. The test sets guide development of the Biosecurity Flag Rubric by providing targets in need of principled categorization, and the Biosecurity Flag Rubric is in turn applied to improve the test sets by identifying test sequences that can be confidently reassigned into the “Flag” and “No Flag” categories.

Starting with the test sets described above, the Biosecurity Flag Rubric has been developed and the test sets improved through community processes by which members of the SBRC propose and review improvements to these artifacts. These processes have been applied by the community of SBRC members to refine the initial categorization of test set sequences into an improved definition of sequences of concern consisting of a theoretical definition embodied in the Biosecurity Flag Rubric and an operational definition embodied in a collection of test set sequences with a lower number of disputes.

### 3.1 Community processes

**FIGURE 4:**
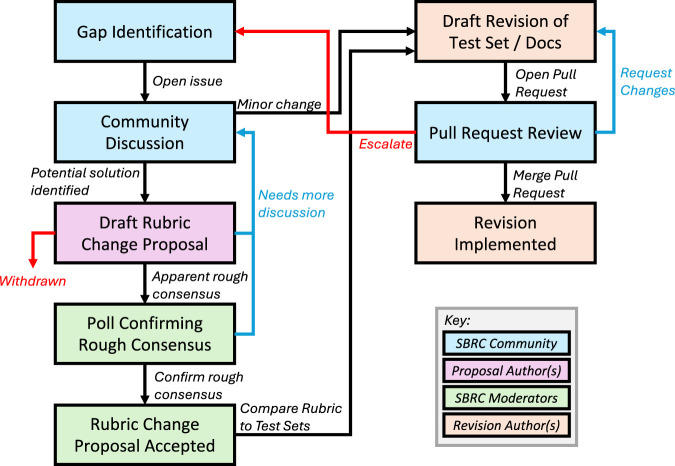
The SBRC revises the Biosecurity Flag Rubric and makes meta-level decisions through a process in which community members identify gaps and discuss potential solutions that are then formalized as Rubric Change Proposals and adopted through IETF-style rough consensus (Resnick, 2014). Community members then compare the Biosecurity Flag Rubric to test sets and documentation to identify areas in need of revision, then implement those revisions using a pull request and review process modeled on standard agile software development practices.

The Biosecurity Flag Rubric is created and maintained through a community proposal process modeled on established practices used by other standards communities (Figure 4). In particular, the SBRC embraces decision-making based on the highly successful Internet Engineering Task Force (IETF) rough consensus process (Resnick, 2014), using proposal templates inspired by the Python community (Warsaw et al., 2000), and polls to affirm consensus modeled on those of the Synthetic Biology Open Language (SBOL) community (SBOL Community, 2025).

Changes to the Biosecurity Flag Rubric and meta-level decision-making regarding community processes are handled via *Rubric Change Proposals* (RCPs) as follows:

- Gap identification: Any member of the SBRC community who notices a gap or other concern needing to be addressed can open an issue for community discussion.
- Community discussion: The issue is discussed and potential solutions are put forth. If the necessary solution involves no real substantive changes (e.g., updating a biological name to track external resources or clarifying the wording used to describe a process), at this point it may be judged by the SBRC moderators as a “minor change” that does not need community review and can be fast-tracked directly to a draft revision and pull request (see below).
- Draft Rubric Change Proposal: Once a viable potential solution has been identified and seems to be gaining support amongst community members, it is made concrete when one or more community members volunteer to draft a formal Rubric Change Proposal (RCP) providing the rationale for the proposed approach, the specific changes that are being proposed, and concrete examples of revisions that will result from adoption of the RCP. All risk assessments in RCPs are required to be science-based and grounded in citations to scientific literature. The community member who identified the gap often also authors the RCP, but there is no necessary relationship. Discussion continues and the RCP is revised as needed until it either appears to have achieved a state of rough consensus (i.e., no significant technical concerns remain that have not been addressed) or else is withdrawn.
- Poll Confirming Rough Consensus: Because much of the SBRC’s work is done asynchronously, it is important to ensure that all community members have the opportunity to review an RCP and raise concerns before it is accepted. For this reason, once the SBRC community moderators assess that an RCP appears to have achieved rough consensus, they run a formal poll with a 1-week notification and comment period, followed by a 1 week period to collect responses. Because RCPs do not proceed to a poll until rough consensus is already believed to have been achieved, the typical outcome is unanimous responses in favor of acceptance. Any vote to the contrary is expected to be accompanied by an expression of specific technical concerns, which are either addressed on the spot if minor or else require the RCP to be returned to the community discussion stage. To date, no RCP has returned to discussion following the opening of a vote, as detailed below in Section 3.2.

Once a proposal has been accepted, it may be applied to revise test sets and documentation. For example, when an RCP was adopted that updated the Biosecurity Flag Rubric to declare that ribosomal RNA genes are “No Flag,” community members searched the test sets for ribosomal RNA sequences and found that some were in other categories. These rRNA sequences could now be understood to be incorrectly categorized according to the definition in the Biosecurity Flag Rubric, and the community members proposed to improve the test set by changing those rRNA sequences to be “No Flag.”

Changes to test sets and documentation are implemented through a faster and more lightweight process based on standard agile software development practices. Any SBRC community member can author a revision to test sets and/or documentation. When the draft revision is ready, they submit it as a “pull request” to merge in the proposed revision (the term “pull request” is taken from software development, where it refers to a request to review and incorporate a proposed change). The draft is then reviewed by other community members, who either approve it to be merged, request changes to improve it, or escalate larger issues for consideration as a potential new RCP.

Taken all together, the result of this process is to ensure that changes to both the Biosecurity Flag Rubric and the test set are acceptable to a broad range of biosecurity stakeholders in the SBRC. The complete specification of this process is attached as Supplementary Material: “RCP 001: Rubric Change Proposals.”

### 3.2 Results of definition refinement

Over the course of 11 months, the SBRC applied these processes to develop version 1.0 of the SBRC Screening Testing Collection, which was released within the SBRC community in September 2025. The community chose to prioritize work on human pandemic-potential viruses as the pathogens typically having the highest potential consequence, so both RCPs and test set improvements during this period focused most heavily in this area.

In total, from the beginning of the process to the 1.0 release, the SBRC community put forward 23 RCPs, of which 17 were accepted and had changes incorporated into the release (implemented via dozens of pull requests), five were accepted and incorporated later (i.e., appearing in the 1.1 release), and one was withdrawn. Of the 17 accepted RCPs, four are process RCPs: one establishing the RCP process itself, one on community governance, and two establishing criteria for evaluating risk. The other 13 are Biosecurity Flag Rubric RCPs: one establishing the initial rubric, five addressing human pandemic-potential viruses, two identifying toxin genes, one identifying certain bacterial virulence factors, and four identifying classes of “No Flag” low-risk sequences.

To date, the observed polling behavior on RCPs has been consistent with the intended operation of the rough consensus process. The sole withdrawn RCP was withdrawn by its author before the vote began based on technical concerns raised during the notification and comment period. Of the others, 18 were adopted unanimously, while the remaining four each had a single contrary vote requesting to address a readily resolved concern: one asked for three extra days to study the proposal, one asked for a discussion to affirm common understanding, and two asked for a minor wording change to better align the specification in the RCP with the expressed intent. Together, these results are consistent with the poll acting as an attention-focusing and quality control process to positively affirm that the community has already reached a mutually satisfactory working consensus.

**FIGURE 5:**
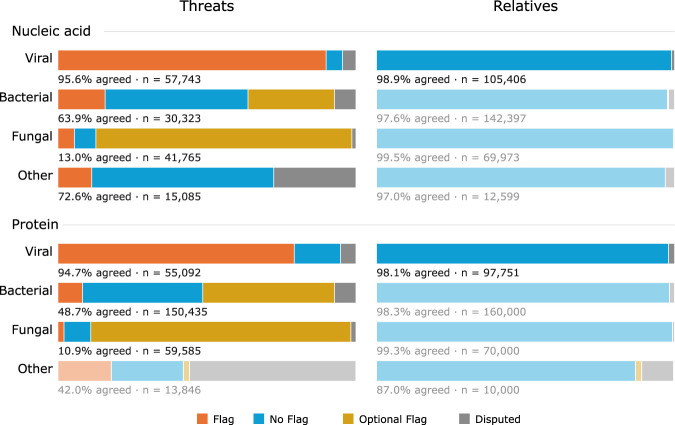
Categorization of test set sequences in SBRC Screening Testing Collection version 1.0, with the “Agreed” category being defined as the sum of “Flag” and “No Flag” results. Collections that were not modified from the initial categorization are indicated by lighter coloration. Exact values and combined statistics are provided in Supplementary Material: “Test Set Statistics.”

**FIGURE 6:**
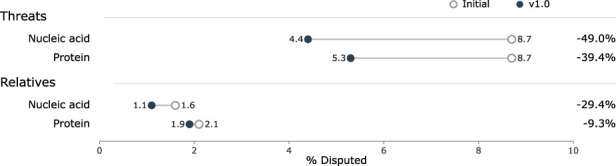
Reduction in “Disputed” sequences for viral test sets in SBRC Screening Testing Collection version 1.0 relative to initial categorization. Non-viral test sets and reduction in “Optional” sequences are omitted because none showed > 2% reduction. Additional values and combined statistics are provided in Supplementary Material: “Test Set Statistics.”

The result of these activities is a Biosecurity Flag Rubric that defines sequences of concern with respect to human pandemic-potential viruses (8 of the 13 viral clusters), key classes of low-risk genes, and controlled toxins. The theoretical definition in the Biosecurity Flag Rubric was applied to the test sets by searching for sequences whose categories did not match the rubric and changing their categories to be the ones specified by the rubric. These rubric-based updates resulted in significant improvements in categorization of test set sequences. The primary area of improvement is in the viral test sets, as shown in Figures 5, 6. This is as expected from the community’s choice of priorities: the five RCPs addressing human pandemic-potential viruses drove a 44.3%, reduction in the total number of “Disputed” sequences for viral threats and an 18.1% reduction for their relatives. Combined with small reductions in “Disputed” sequence counts for other kingdoms from the other RCPs, this resulted in an overall reduction in “Disputed” sequences of 10.7% across the test set collection as a whole. There were also minor improvements in the number of “Optional Flag” sequences, but these were not a significant focus of effort (being a lower priority than disputes) and the overall reduction was only 0.3% (Supplementary Material: “Test Set Statistics.”).

These improvements have since propagated from these test sets back to the biosecurity screening systems participating in the SBRC. Since these changes followed the community processes in Figure 4, the maintainers of each biosecurity system have accepted the rubric definitions and accompanying test set changes (e.g., gene PleD in *R. prowazekii* should not be flagged, while Araraquara virus should be flagged) and updated their systems accordingly.

## 4 Discussion

The SBRC has produced a concrete “sequence of concern” definition, taking the form of a theoretical definition in the Biosecurity Flag Rubric coupled with an operational definition in the collection of test set sequences. Critically, this definition is known to be both acceptable to a broad range of biosecurity stakeholders and compatible with current industrial biosecurity screening best practices, coupled with a process for continuing to improve and maintain this definition over time. These results thus provide a solid foundation on which to build other components of effective biosecurity screening, including proficiency testing, certification, government regulation, and legal enforcement. Because the process grounds out in science-based risk assessment rather than regulation and is developed by a growing international community, it also offers a rendezvous point for international harmonization of standards and regulations. This is particularly timely given the current governmental efforts to develop nucleic acid screening regulations in the United States, United Kingdom, European Union, and other countries. Screening tool providers expect to be held to these definitions are have updated their systems accordingly.

Beyond the work reported here, the SBRC has continued to develop the Biosecurity Flag Rubric and test sets. The priorities selected by the community for 2026 are:

- Expansion of the rubric and dispute resolution to the full range of bacterial and fungal pathogens on the Australia Group lists,
- Extension of the rubric and test sets to high-risk pathogens not currently regulated,
- Function-based criteria for risk assessment, and
- Handling differences in regulation (but not risk) between different jurisdictions.

The SBRC is also developing systematic tests for screening evasion threats such as AI-assisted redesign of proteins (Wittmann et al., 2025), splitting-based obfuscation (Tayouri et al., 2025), and order fragmentation (Edison et al., 2026; Wittmann et al., 2026). The SBRC is also actively expanding its membership to incorporate additional subject-matter experts and stakeholders from a broader range of countries, with the aims of deepening the expertise available for science-based risk assessment (especially with respect to pathogens with limited geographic distribution) and increasing the opportunities for international harmonization.

It is also important to note that this work represents only one aspect of implementing effective enforceable biosecurity standards. Directly complementary to standards for defining sequences of concern is the need for customer screening standards that address the question of how to determine whether a customer should be given access to a sequence of concern (Mackelprang et al., 2025), including questions of customer pre-approval, verification, and the handling of “grey area” cases. Likewise, both governments and non-governmental organizations need to cooperate to utilize these standards to implement systems of certification, regulation, and legal consequences that can drive universal adoption of effective biosecurity screening practices by nucleic acid providers. Finally, the growing complexity of biotechnology and increasing capabilities of AI also point towards a need for biosecurity screening to be adopted more broadly across the whole of the bioeconomy at other points of potential intervention such as biological design tools, AI-assisted protein engineering software, and laboratory information management systems (LIMS), as well as Large Language Models (LLMs), agentic AI systems, and other general purpose tools with the potential to uplift biological capabilities.

## Data Availability

The datasets presented in this article are not readily available because they are part of a curated research resource and are not publicly available. Access is granted to qualified organizations under licensing terms that mitigate potential information hazards and that support the maintenance and long-term sustainability of the data collection. Further information about the data and associated licensing terms is available at https://sbrc.bio/. Requests to access the datasets should be directed to SBRC Moderators (moderators@sbrc.org).
